# Tumor-informed ctDNA assessment as a valuable prognostic and predictive biomarker in diffuse large B-cell lymphoma

**DOI:** 10.3389/fonc.2024.1407003

**Published:** 2024-07-29

**Authors:** Mayur Narkhede, Sarah Tomassetti, Madiha Iqbal, Antony Tin, Samuel Rivero-Hinojosa, Giby V. George, Hayley Widden, Ryan Benrud, Meenakshi Malhotra, Angel Rodriguez, Minetta C. Liu

**Affiliations:** ^1^ Department of Hematology Oncology, University of Alabama at Birmingham, Birmingham, AL, United States; ^2^ Department of Medicine, Harbor-University of California, Los Angeles (UCLA) Medical Center and The David Geffen School of Medicine at University of California, Los Angeles (UCLA), Los Angeles, CA, United States; ^3^ Mayo Clinic, Blood and Marrow Transplant and Cellular Therapy [Chimeric Antigen Receptor T-cell Therapy (CAR-T)] Program, Jacksonville, FL, United States; ^4^ Oncology, Natera Inc., Austin, TX, United States

**Keywords:** molecular residual disease, treatment response monitoring, circulating tumor DNA, next generation sequencing, surveillance

## Abstract

**Background:**

A novel approach for molecular residual disease (MRD) detection and treatment monitoring is needed in diffuse large B-cell lymphoma (DLBCL) to identify patients with a poor prognosis. We performed a retrospective evaluation of commercial ctDNA testing in patients with stage I-IV DLBCL to evaluate the prognostic and predictive role of tumor-informed ctDNA assessment.

**Methods:**

A personalized and tumor-informed multiplex PCR assay (Signatera™ bespoke mPCR NGS assay) was used for ctDNA detection and quantification.

**Results:**

In total, 50 patients (median age: 59 years; median follow-up: 12.68 months) were analyzed, of which 41 had pretreatment time points with ctDNA detected in 95% (39/41). Baseline ctDNA levels correlated with R-IPI scores and stage. ctDNA clearance during first-line therapy was predictive of improved therapy responses and outcomes (EFS, HR: 6.5, 95% CI: 1.9-22, p=0.003 and OS, HR: 22, 95% CI: 2.5-191, p=0.005). Furthermore, 48% (13/27) of patients cleared their ctDNA following the first cycle of treatment. Patients who cleared their ctDNA, irrespective of their R-IPI score, had superior outcomes compared to ctDNA-positive patients. ctDNA clearance outperformed other factors associated with EFS in multivariate analysis (HR: 49.76, 95% CI:1.1-2225.6, p=0.044). Finally, ctDNA clearance predicted complete response (CR)/no evidence of disease (NED) on average 97 days (range: 0-14.7 months) ahead of imaging/biopsy.

**Conclusion:**

ctDNA testing in patients with DLBCL is predictive of patient outcomes and may enable personalized surveillance, intervention, and/or trial options.

## Introduction

Of the non-Hodgkin lymphomas, diffuse large B-cell lymphoma (DLBCL) is the most common subtype, characterized by genotypic and phenotypic heterogeneity. With a median age at diagnosis of 70 years, DLBCL possesses a cure rate of roughly 50-60% with successful standard first-line therapy using rituximab, cyclophosphamide, doxorubicin, vincristine, and prednisolone (R-CHOP) (1). Patients refractory to first-line therapy may require second-line therapy and/or anti-CD19 CAR T-cell therapy, and those who subsequently relapse or who show progressive disease despite intensive treatment may require bispecific antibodies or non-T-cell mediated therapies (2).

Current risk stratification modalities to predict treatment response include cytogenetic aberrations, cell-of-origin subtyping, the use of the Revised International Prognostic Index (R-IPI) scoring system, and Ki-67 proliferation index (3). Large B-cell lymphomas with chromosomal abnormalities such as *MYC* and *BCL2* translocations (and/or less commonly *BCL6* rearrangement) detected by fluorescence *in situ* hybridization (FISH) are classified as double or triple hit lymphomas, respectively (3). Similarly, DLBCLs can also be categorized according to their cell-of-origin (COO) subtype (germinal center B-cell [GCB] or activated B-cell like [ABC]/non-GCB subtypes), traditionally by gene expression profiling but more commonly by immunohistochemistry using the Hans algorithm (3). Response to standard therapy may be predicted based on this distinction with the GCB-subtype generally portending a better prognosis. Similarly, the R-IPI scoring system uses the following criteria to assign patients to one of three risk-groups: age>60 years, serum lactate dehydrogenase (LDH), Ann Arbor stage III/IV disease, Eastern Cooperative Oncology Group (ECOG) performance status ≥2, and >1 site with extranodal involvement) (4). Finally, Ki-67 is a nuclear nonhistone protein synthesized during cell division, which may be used as an index of proliferation (5). A Ki-67 proliferation index >80% by immunohistochemistry is thought to be associated with poorer overall survival (OS) (3).

More recently, comprehensive genetic analysis by way of whole exome sequencing (WES) has revealed the molecular pathogenesis of DLBCL to be much more complex than previously understood (6–8). However, neither morphological nor molecular subtyping from tumor tissue has been able to accurately predict treatment response and patient outcomes (9). Now established as a prognostic and predictive biomarker across several tumor types, circulating tumor DNA (ctDNA) detection enables the identification of patients with molecular residual disease who are likely to be refractory or relapse following treatment. As a minimally invasive, quantitative blood-based biomarker, ctDNA-based disease assessment provides the advantage of serial monitoring of tumor molecular profile during and after treatment, and real-time quantification of molecular disease burden.

Here, we evaluated the prognostic and predictive role of tumor-informed ctDNA assessment in patients with DLBCL. We report on pre- and post-treatment ctDNA detection rates and the association with event-free survival (EFS)/OS. We also compare ctDNA analysis with prognostication using the R-IPI scoring system and propose that ctDNA dynamics may prove a valuable adjunct for treatment response monitoring in DLBCL.

## Materials and methods

### Study population

**Figure 1:**
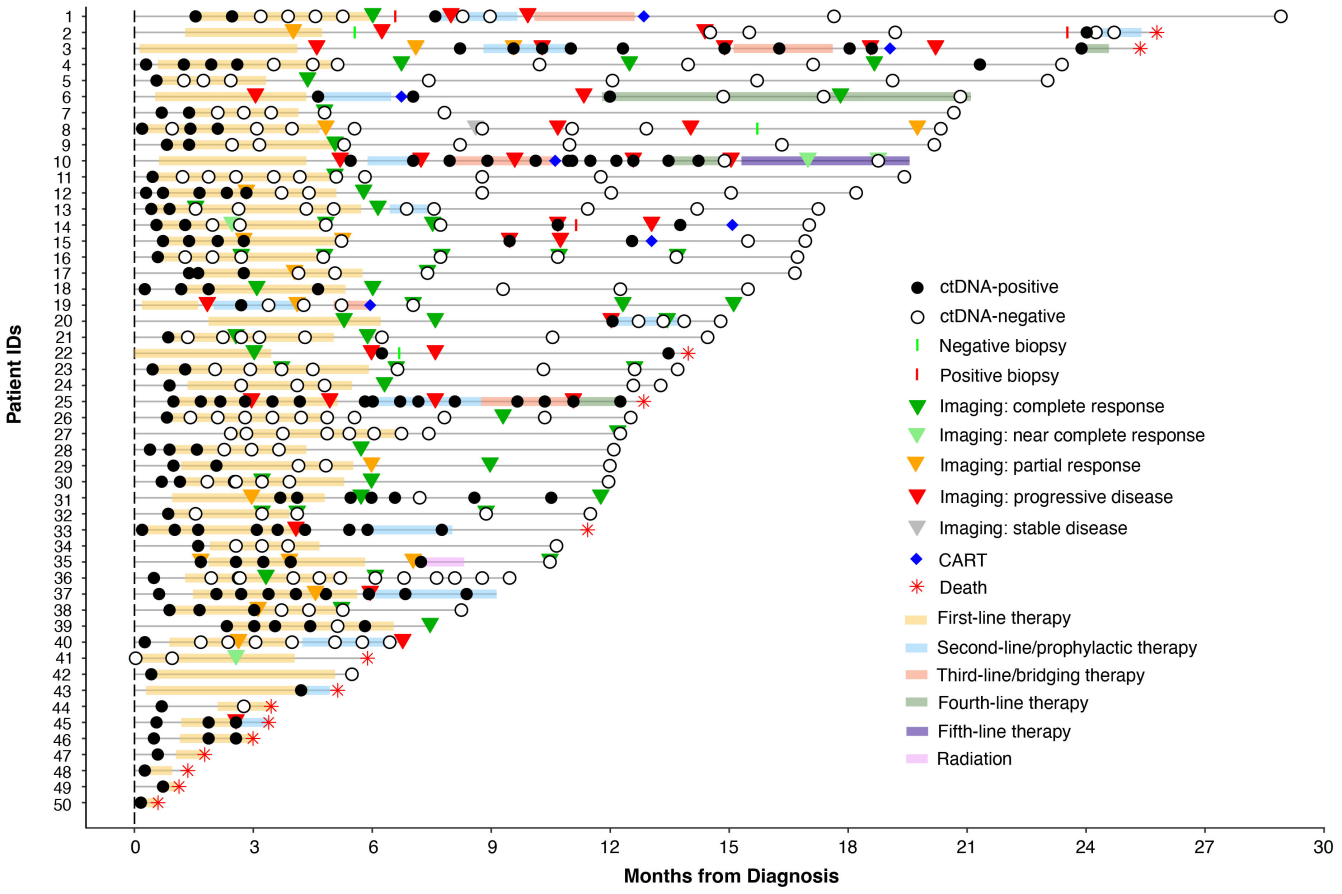
Overview plot showing longitudinal ctDNA status, treatment regimen, and clinical outcomes for patients with stage I-IV DLBCL.

In this retrospective study utilizing commercial ctDNA testing results in patients (N=50) with stage I-IV DLBCL, plasma samples (n=356) were analyzed pre-treatment (baseline), during first-line therapy, during and post-salvage therapy, at the end of treatment, and during surveillance up to the last clinical follow-up from 9/18/20 to 5/20/23. Tumor tissue was collected at initial diagnosis. Blood samples were collected serially at the treating physician’s discretion. Clinicopathologic information was gathered for all patients. All patients received treatment and follow-up in accordance with standard clinical practice and per the oncologist’s discretion. The complete clinical course for all patients and details of therapy are depicted in **Figure 1**. Patient consent was obtained as part of the ordering assay for commercial samples. This retrospective study was approved by the corresponding Ethical and Independent Review Services (protocol# 21-058-GT) and was conducted in accordance with the Declaration of Helsinki.

### Biospecimen collection and processing

Biospecimens were collected and processed as part of clinical testing using a tumor-informed, ctDNA assay. Tumor DNA was extracted from formalin-fixed paraffin-embedded (FFPE) biopsy specimens for all patients. For germline DNA analysis, a single blood sample was collected for each patient in a 6mL EDTA test tube. Blood plasma samples for ctDNA analyses were collected in two, 10mL Streck tubes throughout the patients’ clinical course.

### Customized mPCR-based NGS assay for ctDNA detection

A personalized, tumor-informed, multiplex polymerase chain reaction (mPCR) next-generation sequencing (NGS) assay (Signatera™) was used for the detection and quantification of ctDNA, as previously described (10). Briefly, whole-exome sequencing (WES) was performed on FFPE tumor tissue along with matched normal blood samples. Based on the results of WES, up to 16 patient-specific somatic single nucleotide variants (SNVs) were selected to develop a personalized assay to detect ctDNA in the blood for each patient. Cell-free DNA was extracted from a median of 10 mL of plasma (range: 3-14 ml). Universal libraries were created by end repair, A-tailing, and ligation with custom adapters. Libraries were then amplified by multiplex PCR, barcoded, pooled, and sequenced on an NGS platform. Plasma samples with ≥ 2 SNVs were defined as ctDNA-positive, and ctDNA concentration was reported in mean tumor molecules (MTM)/ml of plasma.

### Genomics analysis

Whole exome VCF files were used to calculate tumor mutational burden (TMB) based on all somatic SNVs/Indels per Mb for all 50 patients as previously described (11). Somatic variants were annotated using the Ensembl Variant Effect Predictor (12). The Maftools R package was used to display mutant genes (13). Only non-synonymous mutations were included; frequently mutated genes in most of the public exome studies were removed from analysis (14). A subanalysis of 39 patients with measurable disease was performed to correlate TMB with ctDNA clearance in response to rituximab-based immunotherapies. The prevalence of driver mutations known to be associated with DLBCL was also evaluated.

### Statistical analysis

Statistical significance was assessed using the chi-squared and Fisher’s exact test to compare categorical variables. Survival analyses were performed using the Kaplan-Meier Estimator and Cox method. These analyses were carried out in R version 4.2.2 using packages survminer, survival, and coxph. The primary outcome measure was EFS, defined as the time elapsed since initiation of first line (1L) therapy during which the patient did not experience progression, relapse, death or treatment with salvage therapy. EFS was censored at the last date of follow-up if the patient was alive and without an event. OS was defined as the time elapsed after initiation of primary treatment until death from any cause. Clinical progression and relapse were defined by imaging unless biopsy was available. To account for potential immortal time bias, a landmark analysis was performed 3 weeks after first-line therapy (after cycle 1) to evaluate the association of ctDNA with EFS and OS. Univariate and multivariable models were analyzed using Cox proportional models. For multivariate analysis, age, gender, Ki-67 proliferation index, COO, R-IPI score, stage, baseline ctDNA levels, and ctDNA status were included as covariates. All P-values were based on two-sided testing and differences were considered significant if P ≤ 0.05. Clinical sensitivity and specificity were calculated at the sample level based on ctDNA status during treatment (any line) or post-treatment surveillance using plasma samples collected prior to the clinical measurement by imaging or biopsy (i.e. complete response, partial response, relapse, refractory, death).

## Results

### Patient cohort

Results were collected from testing 356 plasma samples from 50 patients with stage I-IV DLBCL (I:3, II:12, III: 4, and IV: 31; median age: 59 years). Patients were followed up for a median of 12.68 months (inter-quartile range: 7.9-17.7 months). Demographics for the entire clinical cohort are listed in **Table 1**.

**Table 1:** Patient demographics.

Patient characteristics (N=50)	N	%/range
# Male	28	44%
# Female	22	56%
Average age (years)	60	26-84
Median age (years)	59	26-84
Stage at diagnosis		
I	3	6%
II	12	24%
III	4	8%
IV	31	62%
R-IPI score		
Very good (0)	5	10%
Good (1-2)	17	34%
Poor (3-5)	22	44%
Unknown	6	12%
ECOG performance score		
0	26	52%
1	11	22%
2	6	12%
3	3	6%
Unknown	4	8%
Cell of Origin		
GCB	21	42%
non-GCB	22	44%
Unknown	7	14%
Ki-67 status		
≤60	11	22%
61-70	4	8%
71-80	9	18%
81-100	20	40%
Unknown	6	12%
1L Therapy		
R-CHOP based*	42	84%
R-EPOCH based**	4	8%
R-ICE***	1	2%
R-CHP-POLA****	1	2%
Clinical TRIAL	2	4%
ctDNA & Clinical		
Avg # samples/patient	7	1-15
Median length of followup	12.68	7.9-17.7
# of Deaths	14	

*R-CHOP: R, rituximab; C, cyclophosphamide; H, doxorubicin hydrochloride (hydroxydaunomycin); O, vincristine sulfate (oncovin); P, prednisone.

**R-EPOCH: R, rituximab; E, etoposide phosphate; P, prednisone; O, vincristine sulfate (oncovin); C, cyclophosphamide; H, doxorubicin hydrochloride.

***R-ICE: R, rituximab; I, ifosfamide; C, carboplatin; E, etoposide phosphate. Note that C in the R-ICE regimen differs from R-CHOP.

****R-CHP-POLA: POLA, polatuzumab vedotin (Polivy), in addition to R-CHP (excludes oncovin). #, number of.

### ctDNA detection rates at pre-treatment (baseline) time points

**Figure 2:**
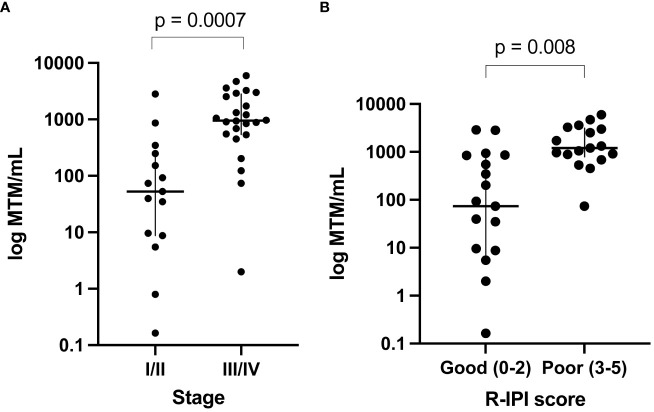
Baseline ctDNA levels were strongly correlated with **(A)** higher stages of disease (III-IV vs. I-II; p=0.0007) and **(B)** poor R-IPI scores (3-5 vs. 0-2; p=0.0008).

Of the 50 patients, 41 had pre-treatment (baseline) time points available and ctDNA was detected in 95% (39/41) of patients. The remaining 5% (2/41) were found to be baseline negative for ctDNA and stayed negative throughout the course of treatment with no evidence of recurrence on subsequent imaging. Since the majority of patients had stage IV disease, median ctDNA detection levels were observed to be high at 534 MTM/ml (range: 0-5957 MTM/ml). In comparing baseline ctDNA levels with other clinicopathological risk factors, high baseline ctDNA levels were observed to be strongly correlated with higher stages of disease (I-II vs. III-IV; p=0.0007) and poor R-IPI scores (0-2 vs. 3-5; p=0.0008) (**Figures 2A, B**) but not with EFS or OS (data not shown).

### ctDNA clearance during first-line therapy and patient outcomes

**Figure 3:**
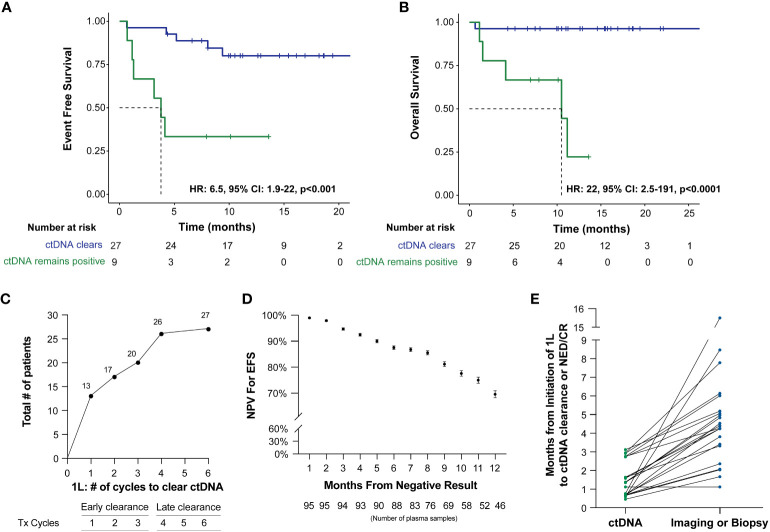
Kaplan-Meier estimates of patients with DLBCL representing ctDNA clearance stratified by **(A)** EFS and **(B)** OS. For this analysis, a landmark was implemented (to account for the potential time bias) 3 weeks after first-line therapy (after cycle 1) to evaluate the association of ctDNA with EFS and OS. **(C)** ctDNA dynamics among patients during the six cycles of 1L therapy. **(D)** NPV for EFS post-1L therapy negative ctDNA result, with NPV >90% for recurrence/death for patients with a negative result within the first 5 months. **(E)** Time of ctDNA clearance vs imaging or biopsy result of NED/CR relative to initiation of 1st line therapy.

ctDNA clearance was defined as the change in ctDNA status (positive to negative) from baseline/during treatment time point to any subsequent time point. Among patients who received first-line therapy and had a ctDNA timepoint available, 75% (27/36) experienced clearance, while 25% (9/36) remained positive. ctDNA clearance was predictive of improved therapy response and patient outcomes (EFS HR: 6.5, 95% CI: 1.9-22, p<0.001 and OS HR: 22, 95% CI: 2.5-191, p<0.0001) (**Figures 3A, B**). Furthermore, 48% (13/27) of patients were found to clear their ctDNA following the completion of the first cycle of treatment and 74% (20/27) by cycle 3, suggesting the majority of the patients clear early (**Figure 3C**). In further evaluating ctDNA clearance during first-line therapy, we found that sustained ctDNA positivity throughout later cycles of treatment (cycles 4-6) was significantly more predictive of a future clinical event than ctDNA positivity during early cycles of treatment (cycles 1-3), with a sample level positive predictive value of 85.7% (6/7, cycle 4-6) vs. 47.4% (18/38, cycle 1-3), respectively, p=0.04 (data not shown). Meanwhile, when evaluating the duration and probability of patients who remained clinically negative after a ctDNA-negative result during 1L therapy, a high negative predictive value (NPV >90%) for relapse/refractory/death for up to 5 months was observed (**Figure 3D**).

Of the 41 patients with imaging and/or biopsy on or after the first line therapy, a total of 70.7% (29/41) patients were found to have achieved CR/NED. Among the 29 CR/NED patients, 26 patients had complementary ctDNA testing available, 88.5% (23/26) of whom achieved ctDNA clearance ahead of NED (as confirmed by PET and/or biopsy) by an average of 97 days (median: 82 days, range: 0-14.7 months) (**Figure 3E**). Of the remaining three, one patient remained negative (#27) at all time points. Patient #18, remained ctDNA-positive up until cycle 5 and was noted to eventually become ctDNA-negative after first-line therapy without any additional intervention (**Figure 1**). Lastly, patient #31 was persistently positive at low levels throughout first-line therapy and remained positive post-therapy (**Figure 1**).

### R-IPI prognostication and its association with ctDNA in predicting patient outcomes

**Figure 4:**
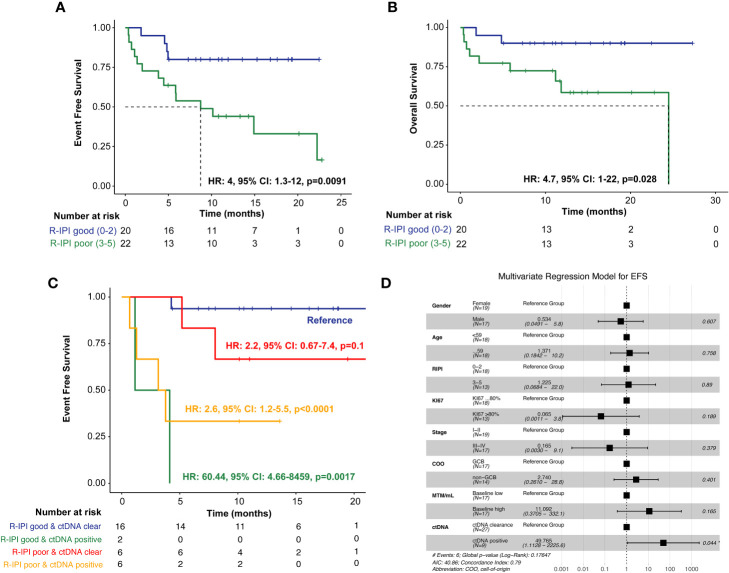
Kaplan-Meier estimates of patients with DLBCL representing R-IPI score stratified by **(A)** EFS and **(B)** OS, **(C)** Kaplan-Meier estimates depicting EFS stratified by R-IPI score and adjunct ctDNA clearance. To account for potential immortal time bias, a landmark analysis was performed 3 weeks after first-line therapy (after cycle 1) to evaluate the association of ctDNA with EFS. **(D)** Multivariate analysis of prognostic factors and their association with EFS, as analyzed by HR, analyzed across the cohort. *P < 0.05.

In evaluating the prognostic utility of the R-IPI model, patients (N=22) with poorer R-IPI scores (3-5) were found to have significantly inferior EFS (HR: 4, 95% CI: 1.3-12, p=0.0091) and OS (HR: 4.7, 95% CI: 1-22, p=0.028) when compared to patients (N=20) with good R-IPI scores (0-2) (**Figures 4A, B**). When utilizing ctDNA status as an adjunct to the R-IPI score, patients who were able to clear their ctDNA on treatment, irrespective of their R-IPI score, were found to have superior outcomes compared to patients who remained ctDNA-positive (**Figure 4C**). In multivariate analysis, we found that ctDNA-positivity significantly outperformed all other risk factors and was highly predictive of inferior EFS (HR: 49.765, 95% CI:1.1128-2225.6, p=0.044) (**Figure 4D**).

### Genomic analysis

**Figure 5:**
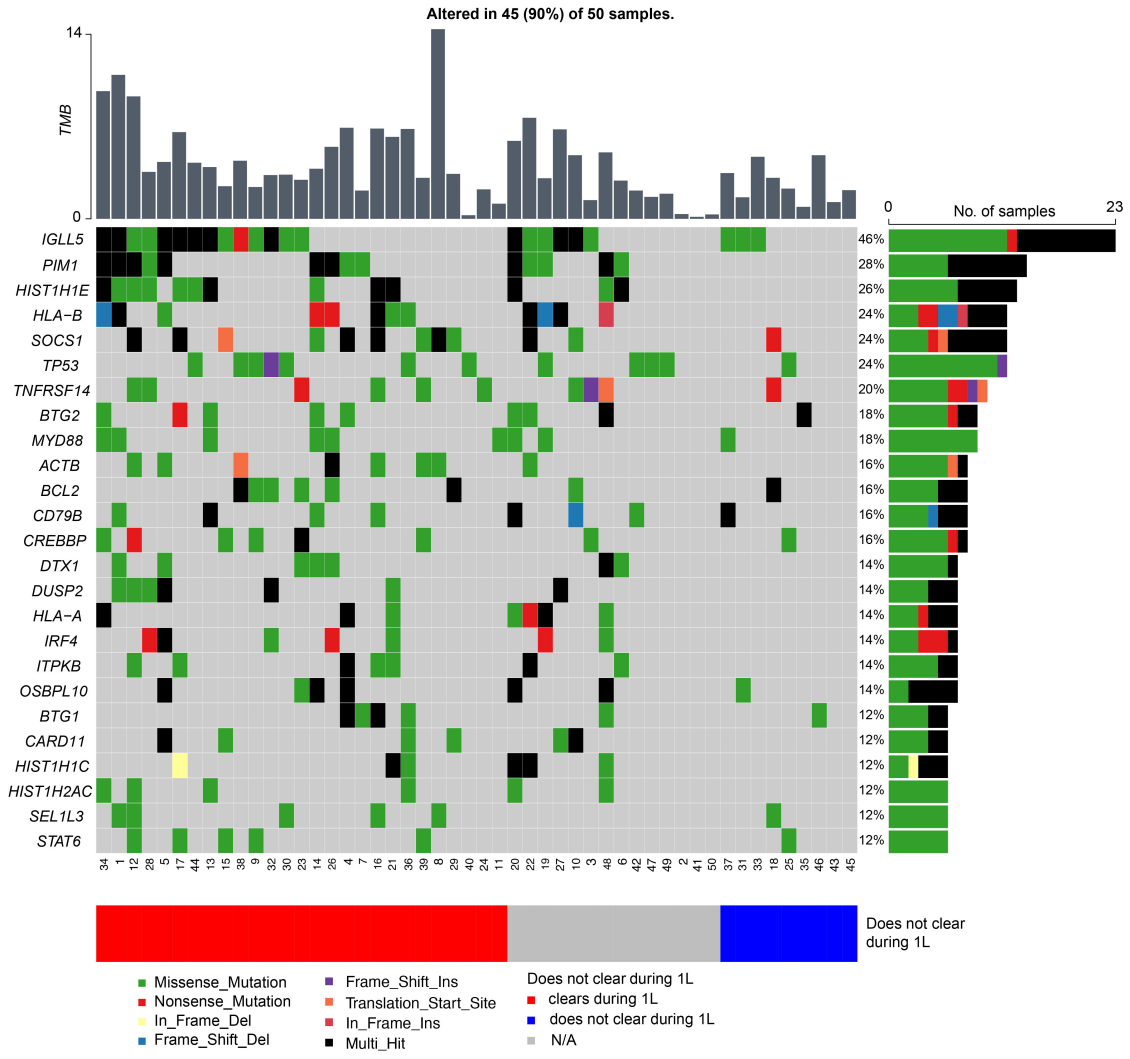
Genetic alterations most frequently observed in patients with DLBCL, with patients represented on the x-axis and mutated genes on the y-axis. The bar graph to the right depicts mutation frequency for each gene within the cohort, while the bar graph above shows the TMB for each patient.

TMB was calculated at a median of 3.37 mutations per megabase (Mb) (range: 0.15–14.38 mutations/Mb) across all 50 patients. In this cohort, the top five mutated genes were *IGLL5* (46%), *PIM1* (28%), *HIST1H1E* (26%), *HLA-B* (24%), *SOCS1* (24%), and *TP53* (24%) (**Figure 5**). When stratified by ctDNA clearance during first-line therapy (irrespective of the cycle) vs never cleared, patients who did not clear their ctDNA were observed to have a lower TMB rate. The average TMB in patients who experienced ctDNA clearance was 4.97 compared to 2.71 in patients who did not clear, p<0.007.

## Discussion

Despite enhanced methods to decode the genomic and clinical heterogeneity of DLBCL, neither subtyped nor risk-stratified interventions have been able to improve patient outcomes (9). Early data has indicated that pretreatment ctDNA is largely reflective of tumor burden and that ctDNA dynamics during and after therapy are predictive of treatment response (15). In our study, we found that pretreatment ctDNA levels can be used as a reliable surrogate marker for tumor burden in DLBCL. Moreover, with an overall clinical sensitivity of 88.8% (24/27) and clinical specificity of 94.1% (32/34), we report that on-treatment ctDNA dynamics are predictive of therapy response and highly prognostic of relapsed/refractory disease.

Using a personalized, tumor-informed ctDNA assay, we explored the significance of baseline and dynamic ctDNA levels for predicting patient outcomes. We found pretreatment ctDNA levels to be strongly correlated with higher R-IPI scores and more advanced stages of disease. Although previous data reported pretreatment ctDNA levels to be prognostic of EFS (15), we observed no correlation between baseline MTM/ml levels and EFS/OS, likely due to the large number of stage IV patients and differences in sampling. Regardless, our data suggest a role for baseline ctDNA levels as a surrogate for tumor burden which could potentially improve pretreatment risk stratification.

ctDNA dynamics, specifically clearance at any point during first-line therapy, was found to be predictive of improved response rate and outcomes (EFS, HR: 6.5, 95% CI: 1.9-22, p<0.0006 and OS, HR: 22, 95% CI: 2.5-191, p<0.0001), compared to patients who remained ctDNA-positive. Specifically, 48% (13/27) of patients were found to clear their ctDNA by cycle 1 of treatment, and 74% (20/27) cleared by cycle 3. Although the majority of patients cleared their ctDNA by the end of the first three cycles of first-line therapy, patients who cleared their ctDNA late showed similar outcomes to those who cleared early [patient level NPV of 96.2% (26/27)]. However, in contrast to pretreatment levels, ctDNA measurement during first-line therapy demonstrated superior stratification of outcomes, underscoring the utility of longitudinal monitoring as a potential predictor of therapy response and recurrence.

ctDNA clearance was observed in 88% of patients ahead of clinical CR/NED findings. Our findings expand on those reported by Roschewski et al (16), who retrospectively evaluated an NGS-based approach for analyzing ctDNA encoding the VDJ immunoglobulin gene rearrangements in patients with previously untreated DLBCL. In the surveillance setting, they found detectable ctDNA to be associated with clinical disease progression with an HR of 228 (95% CI: 51-1022, p<0.0001) compared to patients with undetectable ctDNA. More importantly, we found that when utilizing ctDNA status as an adjunct to the R-IPI score, patients who were able to clear their ctDNA, irrespective of their R-IPI score, were found to have superior outcomes compared to patients who remained ctDNA-positive, highlighting the utility of ctDNA monitoring.

Beyond the primary treatment setting, molecular residual disease (MRD) assessment may be utilized to guide the optimal use of autologous stem cell transplantation (ASCT) in patients with relapsed/refractory (R/R) DLBCL. Merryman et al. used immunoglobulin high-throughput sequencing for MRD evaluation of apheresis stem cell (ASC) samples, post-ASCT peripheral blood mononuclear cells, and plasma samples collected from 159 patients with R/R DLBCL. MRD was detected in 23% (23/98) of ASC samples and was associated with an inferior progression-free survival (PFS) (5-year PFS 13% vs 53%, p<0.001) and poor overall survival (52% vs 68%, p=0.05). ASC MRD positivity was found to be a significant predictor of PFS in multivariable analysis (HR: 3.7, p<0.001), as was a positive plasma MRD result (PFS HR: 3.0, p=0.016), supporting the incorporation of post-ASCT MRD surveillance testing to identify patients at high risk of relapse.

In surveying the molecular landscape of our cohort, we observed the top 25 prevalent mutations were present in 90% (45/50) of the patients (**Figure 5**). Presently, there are several proposed molecular classification systems for DLBCL (17). Chapuy et al. originally described five clusters (C1-C5) based on WES data of 304 *de novo* DLBCL cases (6). Other groups have since published similar molecular classification schemes and subsequent analysis has identified seven possible genetic DLBCL subtypes based on the LymphGen algorithm (7, 8). However, in our study we were unable to identify any molecular subgroups in our cohort, likely due to our limited sample size and lack of RNA-sequencing and gene expression profiling. Moreover, molecular analysis of our relapsed/refractory patients did not reveal any unique genomic characteristics contrary to Liu et al. who cited unique mutations in their relapsed/refractory DLBCL cohort and Meriranta et al. who reported frequent *TP53* loss and *MYC* alterations in this group (18, 19). However, when stratified by ctDNA clearance during first-line therapy (irrespective of cycle) versus never cleared, patients who were not able to clear their ctDNA were observed to have fewer mutations, resulting in an overall lower TMB rate. Although the relationship between TMB and chemotherapy response is uncertain (20), a higher TMB may theoretically enhance tumor chemosensitivity. Chen et al. posited that tumors with higher TMB may induce increased neoantigen production, making them an attractive target for activated immune cells (21). Although further studies are warranted to support this hypothesis, based on our preliminary findings and the ongoing challenges with molecular subclassification (9), we suggest that serial ctDNA assessment may more accurately define recurrence risk in patients with DLBCL.

Our study possesses limitations. Our cohort of patients was small, and our analysis was retrospective, rendering us unable to control for confounders that may have affected patient selection for ctDNA testing. Prospective studies in a larger cohort are needed to confirm the prognostic and predictive significance of ctDNA analysis and may enable further molecular characterization of this heterogeneous entity. Regardless, DLBCL possesses a sizable unmet need due to its rate of relapse and frequent treatment refractoriness.

Overall, our findings suggest that ctDNA analysis may enable accurate baseline estimation of disease burden and reliable dynamic monitoring for prediction of treatment response. Our study demonstrates that ctDNA analysis in DLBCL is feasible and may add clinical value beyond currently established biomarkers.

## Data availability statement

The datasets presented in this article are not readily available because The authors declare that all relevant data used to conduct the analyses are available within the article. Any additional information can be requested from the corresponding author. Requests to access the datasets should be directed to Mayur Narkhede, msnarkhede@uabmc.edu.

## Ethics statement

This study was conducted in compliance with Natera Protocol 21-058, the Declaration of Helsinki, Title 21 of the United States Code of Federal Regulations (CFR) as applicable, Good Clinical Practice (GCP) guidelines, and International Conference on Harmonization (ICH) guidelines. The requirement of informed consent was waived according to 45 CFR 46.116(d) and 45 CFR 46.117(c)(2), respectively.

## Author contributions

MN: Writing – review & editing, Supervision, Project administration, Data curation, Conceptualization. ST: Writing – review & editing, Supervision, Project administration, Data curation, Conceptualization. MI: Writing – review & editing, Supervision, Project administration, Data curation, Conceptualization. AT: Writing – review & editing, Visualization, Software, Project administration, Methodology, Formal analysis. SR: Writing – review & editing, Visualization, Software, Project administration, Methodology, Formal analysis. GG: Writing – review & editing, Writing – original draft, Visualization, Software, Project administration. HW: Writing – review & editing, Project administration, Data curation. RB: Writing – review & editing, Project administration, Data curation. MM: Writing – review & editing, Writing – original draft, Visualization, Software, Project administration. AR: Writing – review & editing, Supervision, Project administration. ML: Writing – review & editing, Supervision, Project administration.
